# Asymptomatic Cryptogenic Brain Abscess: A Case Report

**DOI:** 10.7759/cureus.26644

**Published:** 2022-07-07

**Authors:** Raquel K Gil, James Yu, Guillermo Izquierdo-Pretel

**Affiliations:** 1 Medical Student, Herbert Wertheim College of Medicine, Florida International University, Miami, USA; 2 Internal Medicine, Herbert Wertheim College of Medicine, Florida International University, Miami, USA

**Keywords:** alcohol use disorder, brain abscess, cryptogenic, asymptomatic, case report

## Abstract

We report a case of a cryptogenic brain abscess in a 48-year-old immunocompetent male who was admitted for acute alcohol intoxication and a fall. A computed tomography scan (CT) of the brain showed a 10.5mm solitary mass in the parieto-occipital lobe. After his initial symptoms were resolved, there were no acute neurological or systemic symptoms. Due to the incidental CT finding, an extensive work up was conducted, including a brain biopsy, which resulted in a surprising diagnosis of brain abscess with no identified source of infection. He was treated with cefepime, metronidazole, and vancomycin. Literature review was done through PubMed searching for cases of cryptogenic brain abscesses with no neurologic symptoms. The review resulted in cryptogenic cases but no cases of asymptomatic cryptogenic brain abscesses.

## Introduction

A brain abscess is defined as a focal infection of the brain that begins as a localized area of cerebritis resulting in a collection of pus surrounded by a well-vascularized capsule [1]. Based on various studies, brain abscesses are most common in men around 40 to 50 years old [2-4]. The “triad” of symptoms associated with brain abscesses are fever, headache, and altered mental status. However, only a small minority of patients (~15-20%) present with all of these three symptoms [5]. The most common presenting symptom for a patient with a brain abscess is headache, which is seen in 50-93% of patients [1,3-5]. Based on the literature, other symptoms that patients may present with include nausea, vomiting, seizures, neck stiffness, hemiparesis, and visual disturbance [1,3,4]. The symptoms can differ based on the location of the brain abscess causing a variety of focal neurological deficits [4]. Despite the variability in presentation of the symptoms, it is rare for patients to completely lack manifestations, including generalized systemic signs of infection such as fever and erythrocyte sedimentation rate (ESR) elevation. A literature review yielded no studies or case reports reporting asymptomatic patients diagnosed with a brain abscess. Here, we present a case report of an asymptomatic cryptogenic brain abscess.

## Case presentation

A 48-year-old immunocompetent male presented to an emergency department in Miami acutely intoxicated and complaining of chest pain. Patient also reported falling outside the hospital hitting the left side of his head on the floor. He has a history of primary hypertension, alcohol abuse, cocaine abuse, chronic hepatitis C, and unspecified onset of psychosis, anxiety, and depression. He denied a history of IV drug abuse or any other medication use. On the initial examination he was found to be tremulous with mild soft tissue swelling lateral to the left orbit. The remainder of the examination was negative, including no evidence of track marks. He had no focal neurological deficits, headache, nausea, vomiting, drowsiness, dizziness, blurry vision, fevers, or chills. His electrocardiogram (ECG) and troponin were both negative. The ethanol levels were 333 (normal is < 50). Table 1 shows patient's labs upon admission to the hospital. 

**Table 1:** Labs Upon Admission WBC: white blood cell, MCV: mean corpuscular volume, BUN: blood urea nitrogen, AST: aspartate transaminase, ALT: alanine aminotransferase, GFR: glomerular filtration rate, HDL: high-density lipoprotein, CRP: C-reactive protein, ESR: erythrocyte sedimentation rate, HCV: hepatitis C virus

Complete Blood Count (CBC)		Normal
WBC	4.9	4.5-11 K/uL
Hemoglobin	14.4	Men: 13.2-16.6 g/dL
Hematocrit	43.6	Men: 38.3-48.6%
MCV	93.2	80-100 fl
Platelet	470	150-450 x 10^9^/L
Absolute Neutrophil	2.8	2.5-6 x 10^9^/L
Absolute Lymphocyte	1.4	1-4.8 x 10^9^​​​​​​​/L
Absolute Monocyte	0.5	0.2-0.95 x 10^9^​​​​​​​/L
Absolute Eosinophil	0.06	0-0.5 x 10^9^​​​​​​​/L
Absolute Basophil	0.04	0-0.3 x 10^9^​​​​​​​/L
Absolute Immature Granulocyte	0.02	0.015-0.085 x 10^9^​​​​​​​/L
Complete Metabolic Panel (CMP)		
Glucose	110	60-100 mmol/L
Sodium	141	135-145 mEq/L
Potassium	4.8	3.6-5.2 mEq/L
Chloride	102	96-106 mEq/L
CO2	29	23-29 ppm
Anion Gap	10	8-12 mEq/L
Osmolality	279	275-295 Osm/kg
BUN	3	6-24 mg/dL
Creatinine	1	0.7-1.3 mg/dL
Calcium	9.3	8.5-10.5 mg/dL
Total Protein	7.9	6-8.3 g/dL
Albumin	4.6	3.4-5.4 g/dL
Total Bilirubin	0.4	0.1-1.2 g/dL
AST	112	8-48 IU/L
ALT	177	7-56 IU/L
Alkaline Phosphatase	99	30-120 IU/L
GFR	89	>60 mL/min
Magnesium	1.3	1.7-2.2 mEq/L
Cholesterol	183	<200 mg/dL
HDL	69	Men: 45-70 mg/dL
Triglycerides	53	<150 mg/dL
Troponin	< 0.012	0-0.04 ng/mL
Hemoglobin A1c	5%	<5.7 g/dL
CRP	< 0.5	0.8-1 mg/L
ESR	57	0-15 mL/h
Other		
Ethanol	333	<50 mg/dL
HCV RNA	1,372,633	“Not detected”
Urinalysis	Normal	Normal

**Figure 1:**
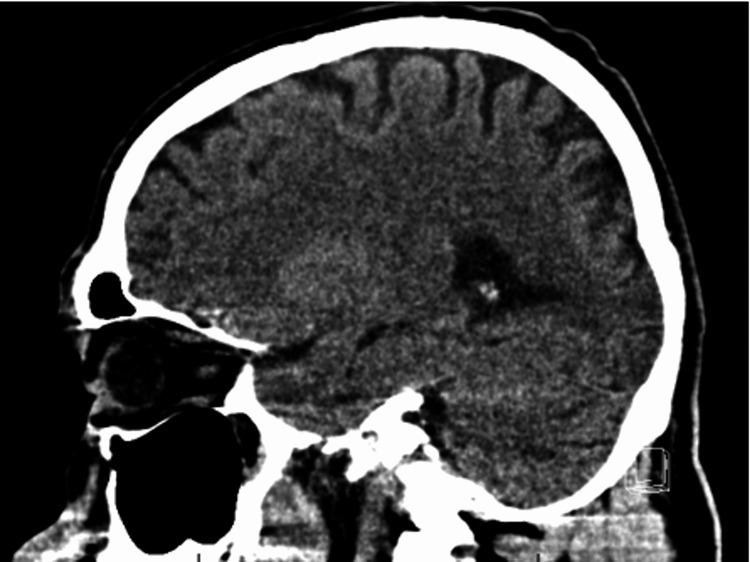
Normal brain CT without contrast (sagittal view)

**Figure 2:**
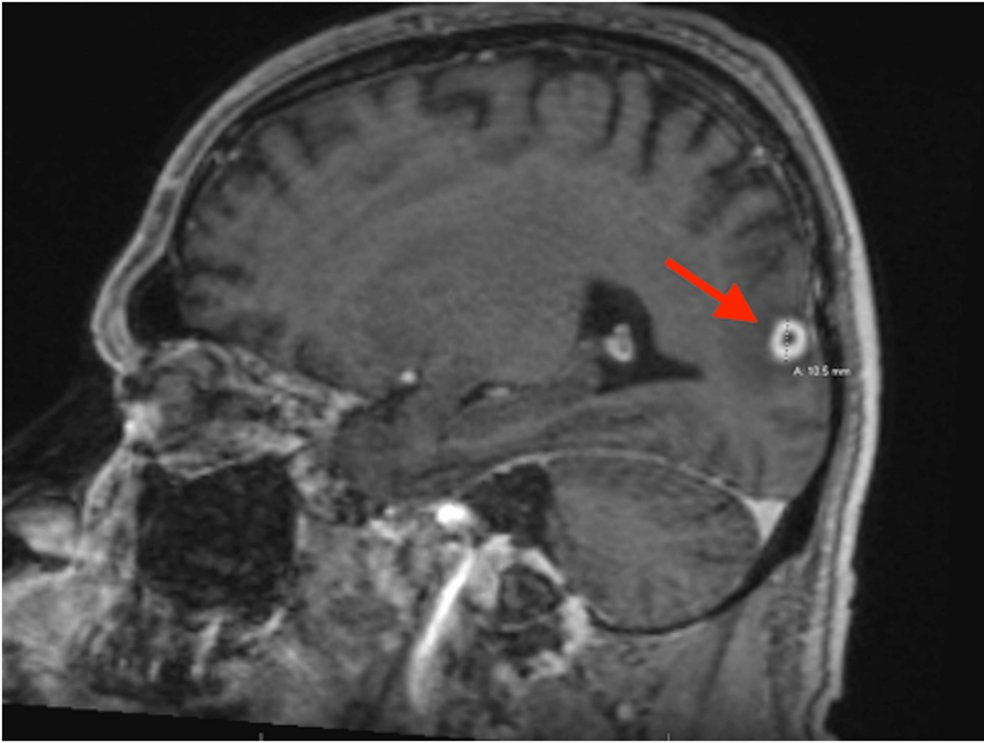
MRI of the brain with 10.5mm lesion in parieto-occipital lobe (FLAIR, sagittal view)

**Figure 3:**
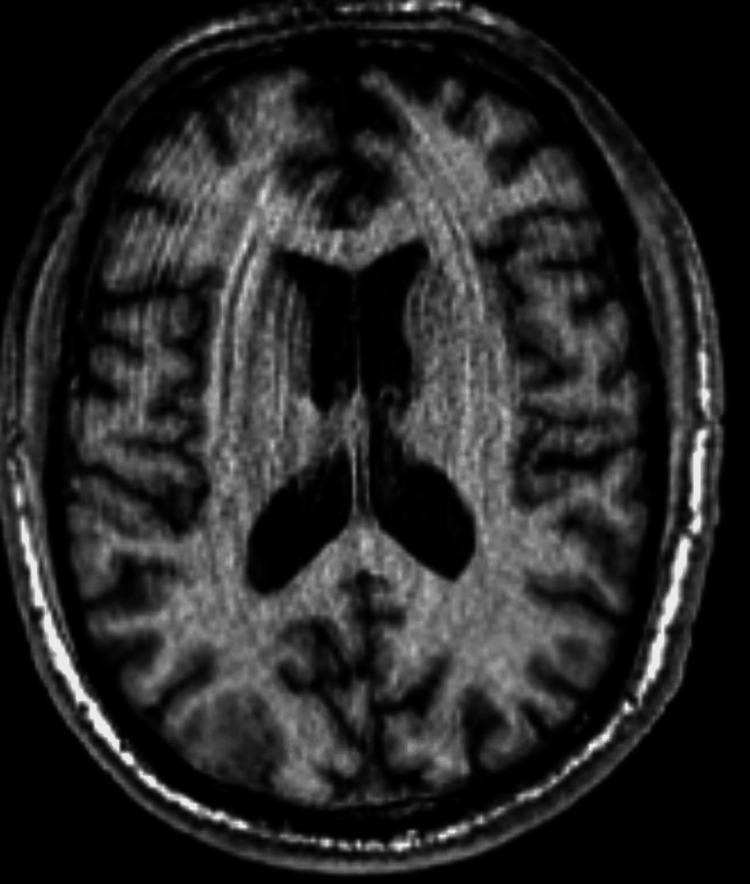
MRI with contrast (no significant findings)

The patient received a CT scan one month prior to this admission (due to another fall believed to be associated with alcohol use) and no lesion was seen (Figure 1). A brain MRI without contrast was ordered showing a 10.5 mm lesion in the parieto-occipital lobe (Figure 2). This was believed to represent an abscess or metastasis. A contrast-enhanced MRI was ordered at the same time and showed no significant abnormalities (Figure 3). 

A CT of the abdomen, pelvis, and chest did not reveal any cancer. Investigation for an infectious process was done for HIV, methicillin-resistant Staphylococcus aureus (MRSA), bacterial culture, acid fast bacilli culture, mycology culture, and viral culture for herpes which were all negative.

After six days of admission, he was asymptomatic and cleared for surgery as infectious disease recommended a biopsy of the lesion for appropriate treatment. He was taken for drainage, biopsy, and culture of the mass by Neurosurgery. It was noted that the fluid drained was grossly purulent and the patient was then empirically started on 2g cefepime intravenous three times per day (TID), 500 mg metronidazole oral (PO) TID, and 2g vancomycin intravenous two times per day (BID). Culture from the brain biopsy showed no growth four days after surgery and the pathology report identified reactive gliosis with chronic inflammation and micro abscesses leading to a final diagnosis of an asymptomatic cryptogenic brain abscess.

The patient denied having fever, headaches, changes in vision, seizures, nausea, vomiting, or hemiplegia in the month prior to diagnosis. As no primary source of infection was identified, we consulted Dentistry and Oral Maxillofacial Surgery (OMFS) as cavities may be a potential source of primary infection. They noted that the patient had two to three potential cavities on the left side of his mouth. A Panorex (x-ray providing view of upper and lower jaws, teeth, temporomandibular joints, and sinuses) showed a left upper premolar cavity and periapical lucency/abscess. Dentistry and OMFS determined that the cavities were not acutely infected due to lack of pain, redness, or drainage surrounding the cavities and their opinion was that it is unlikely to be the primary source of infection. Patient also had a transthoracic echocardiogram done which returned normal results, ruling out pulmonary arteriovenous fistula or valvular mass as a potential primary source of infection. The patient opted against antibiotic treatment, including antibiotics, and was discharged against medical advice. He completed only 10 days of proposed treatment. The patient understood the serious complications of untreated abscesses including coma, intraventricular rupture, ventriculitis, and rupture of abscess potentially leading to death [6].

Table 2 shows a timeline of events during this patient's admission to the hospital. 

**Table 2:** Timeline of Major Events

Date	
2/19	Brain CT w/o contrast done due to altered mental status and found to be normal
3/11	Presents to ED acutely intoxicated for chest pain and fall. CT identifies brain lesion in right parieto-occipital lobe. CXR and EKG normal
3/12	MRI without contrast shows vasogenic edema but no ischemia or hemorrhage
3/13	MRI with contrast done favoring abscess MRI MRV without contrast indicates likely abscess
3/14	2D echo with doppler shows no abnormality CT of abdomen, chest, and pelvis shows no abnormality
3/15	Infectious Disease recommends biopsy followed by vancomycin and cefepime
3/16	Stress test for chest pain with normal results
3/18	Brain biopsy done, large amounts of pus in lesion found
3/20	Psychiatry evaluates and determines patient is fit for outpatient management
3/24	Panorex of mandible done showing premolar and periapical lucency/abscess
3/25	Oral Maxillofacial Surgery and dentistry consults ruling out dental abscess as primary site of infection

## Discussion

The presented case seems to be the first report of a patient with a brain abscess without symptoms. The surprising diagnosis was determined by a pathological report obtained by a craniotomy with brain biopsy. Additional limitations include the inability to determine the infectious source. This makes it difficult to ascertain what the principal risk factors for cases like this might be and it denies us insight into what empiric therapies would most likely be efficacious. Above we presented laboratory data with testing for confounding medical issues (HIV, diabetes, hepatitis).

Brain abscesses occur most commonly in men, and often the source of infection and a causative organism are not identified, with 4.6-43.4% of cases classified as cryptogenic [3], possibly due to early antibiotic initiation. Typical primary sources of infection include otitis, mastoiditis, sinusitis, neurosurgical procedures, trauma, and hematogenous dissemination (endocarditis, dental infection, or pulmonary abscess) [2]. However, up to 43% of patients found throughout various systematic reviews were diagnosed with a brain abscess but had no primary source of infection identified [3,7,8]. Patients included in the studies we reviewed were all determined to be immunocompetent.

The symptomatology is highly variable but typically begins seven to 25 days prior to presentation and generally includes headache, fever, change in mentation, focal neurological symptoms, seizures, nausea, and vomiting. The radiographic findings of brain abscesses are similar to those seen in this case with a lesion found on CT and MRI. The location of the abscess can vary based on the location of the primary infection, so it is not uncommon to find an abscess in the parieto-occipital lobe as seen in this patient. Given the sensitivity of the brain, empiric treatment should not be delayed and is usually continued even when cultures are negative [4]. Empiric regimens often seek to cover common organisms involved in oral and facial infections as well as anaerobes from the oral cavity. Additional coverage may be warranted if a hematological source or nosocomial source is suspected. A common regimen is cefotaxime and metronidazole with or without vancomycin if MRSA is suspected. The most common causes of brain abscesses are Staphylococcus aureus, Staphylococcus epidermidis, and Enterobacteriaceae (Pseudomonas aeruginosa is the most common within this group) [3]. Complications of untreated brain abscess can be severe and include outcomes such as focal neurological deficits and death [6]. In cases with cryptogenic abscesses, they should still be treated empirically with a cephalosporin, metronidazole, and vancomycin. Additionally, stereotactically guided aspiration is shown to be effective for management of brain abscesses [7].

In the presented case, we cannot be certain about the onset and course of the infection, although normal imaging from one month prior to admission does suggest the infection began within the last few weeks at most. The absence of this information makes it impossible to characterize the course of infection and how long it went undiagnosed and untreated. It is possible that the patient's habits, including alcohol use and a subtle history of psychiatric disease, could mask subtle neurological or systemic signs of infection. It is important to note that this is the main confounding variable of this study as there was no way for us to determine whether the patient did have underlying neurologic symptoms that were not evident based on his other history. Family members were unavailable for contact for further insight into any potential changes.

## Conclusions

Asymptomatic cryptogenic brain abscesses have not been described in the medical literature making this case report a unique presentation. Given the lack of studies on asymptomatic brain abscesses, we would like to call attention to the possibility that there may be patients who either have no obvious symptoms associated with an underlying brain abscess or have other factors (e.g., substance abuse and psychiatric disorders) masking the most common presenting symptoms. The absence of symptoms makes it difficult to assess the severity of the infection and determine the intensity of treatment, but dealing with CNS infections, it is recommended to treat aggressively and as soon as possible. The treatment plan on what is considered the gold standard (cephalosporin, metronidazole, and vancomycin) is applicable to cases of cryptogenic brain abscesses, discovered incidentally or otherwise.

## References

[1] Sonneville R, Ruimy R, Benzonana N (2017). An update on bacterial brain abscess in immunocompetent patients. Clin Microbiol Infect.

[2] Carpenter J, Stapleton S, Holliman R (2007). Retrospective analysis of 49 cases of brain abscess and review of the literature. Eur J Clin Microbiol Infect Dis.

[3] Patel K, Clifford DB (2014). Bacterial brain abscess. Neurohospitalist.

[4] Song L, Guo F, Zhang W, Sun H, Long J, Wang S, Bao J (2008). Clinical features and outcome analysis of 90 cases with brain abscess in central China. Neurol Sci.

[5] Huang J, Wu H, Huang H, Wu W, Wu B, Wang L (2021). Clinical characteristics and outcome of primary brain abscess: a retrospective analysis. BMC Infect Dis.

[6] Roche M, Humphreys H, Smyth E (2003). A twelve-year review of central nervous system bacterial abscesses; presentation and aetiology. Clin Microbiol Infect.

[7] Zhang C, Hu L, Wu X, Hu G, Ding X, Lu Y (2014). A retrospective study on the aetiology, management, and outcome of brain abscess in an 11-year, single-centre study from China. BMC Infect Dis.

[8] Alvis Miranda H, Castellar-Leones SM, Elzain MA, Moscote-Salazar LR (2013). Brain abscess: current management. J Neurosci Rural Pract.

